# Natural history and impact of *Giardia lamblia* on child growth attainment and associated pathway-specific biomarkers in a Nicaraguan birth cohort

**DOI:** 10.1371/journal.pntd.0013734

**Published:** 2026-05-15

**Authors:** Lester Gutiérrez, Nadja A. Vielot, Yaoska Reyes, Roberto Herrera, Christian Toval-Ruiz, Javier Mora, Michael B. Arndt, Rebecca Barney, Robert K.M. Choy, Filemón Bucardo, Samuel Vilchez, Sylvia Becker-Dreps, Luther A. Bartelt

**Affiliations:** 1 Centro de Investigación de Enfermedades Tropicales (CIET), Facultad de Microbiología, Universidad de Costa Rica, San José, Costa Rica; 2 Department of Family Medicine, University of North Carolina at Chapel Hill, Chapel Hill, North Carolina, United States of America; 3 Department of Environmental Sciences and Engineering, University of North Carolina at Chapel Hill, Chapel Hill, North Carolina, United States of America; 4 Universidad Tecnológica La Salle, León, Nicaragua; 5 Laboratory of Helminthology, Faculty of Microbiology, University of Costa Rica, San José, Costa Rica; 6 Atria Health and Research Institute, Seattle, Washington, United States of America; 7 PATH, Seattle, Washington, United States of America; 8 Department of Microbiology and Immunology, University of North Carolina at Chapel Hill, Chapel Hill, North Carolina, United States of America; 9 Department of Epidemiology, University of North Carolina at Chapel Hill, Chapel Hill, North Carolina, United States of America; 10 Center for Gastrointestinal Biology and Disease and the Departments of Medicine, University of North Carolina at Chapel Hill, Chapel Hill, North Carolina, United States of America; 11 Division of Infectious Diseases, Department of Medicine, University of North Carolina at Chapel Hill, Chapel Hill, North Carolina, United States of America; undefined, UNITED STATES OF AMERICA

## Abstract

**Background:**

*Giardia lamblia* (*Giardia*) is one of the most common intestinal parasitic infections globally, with an estimated 280 million symptomatic infections annually. In children from low- and middle-income countries (LMICs), *Giardia* is highly prevalent and has been associated with loss of intestinal barrier function, nutrient-metabolic dysregulation, and linear growth impairment, but specific mechanisms linking *Giardia* to these outcomes remain poorly understood.

**Methods and results:**

We used data and samples from a subset of 76 children in a longitudinal birth cohort in Nicaragua to evaluate the natural history and geospatial distribution of *Giardia* infections, child growth outcomes (weight-for-age [WAZ] and length-for-age [LAZ] z scores), and relationships with established biomarkers of inflammation, intestinal damage, and growth-signaling. During the first 36 months of life, we tested 2,305 stools (1,903 surveillance stools and 402 diarrheal stools) for *Giardia* by qPCR. The incidence of *Giardia*-positive stools was 59.6 per 100 child-years. Any detection of *Giardia* was associated with a reduction in LAZ at 36 months of life (β:-0.16, *P* = 0.042). This effect increased when considering persistent or recurrent *Giardia* detections (β:-0.26, *P*=<0.001) as well as living in a high-density *Giardia* detection area (β:-0.44, P=<0.001). Among intestinal markers, *Giardia* was associated with lower median fecal neopterin (a marker of chronic intestinal T cell activation) at 24 and 36 months of age. Among serum systemic biomarkers measured at 24 months, *Giardia* detections were associated with indicators of intestinal epithelial cell damage (higher median Intestinal Fatty Acid Binding Protein (*P* = 0.002) and Anti-FliC IgA (P = 0.033), and reduced growth-signaling hormone (lower median Insulin-like Growth Factor (IGF-1) (*P* = 0.005).

**Conclusion:**

*Giardia* detection was negatively associated with linear growth in an exposure-dependent manner. Simultaneously, *Giardia* was associated with diminished serum growth-signaling hormones. Patterns of serum and fecal intestinal biomarkers suggest that *Giardia-*mediated epithelial disruption is dissociated from markers of intestinal inflammation.

## Author summary

Although *Giardia* was recognized as a pathogen by the World Health Organization in 1981, its long-term effects on child health, particularly in endemic areas, remain poorly understood. One major challenge is that most *Giardia* infections are asymptomatic and go unreported, leading to an underestimation of not only its true burden but also its association with child health outcomes. While a link between *Giardia* and impaired linear growth is becoming clearer in large prospective child cohorts, considerable variability persists in the strength and consistency of this association across studies. Moreover, the mechanistic pathways underlying long-term sequelae in children remain elusive. To contribute to this field, we leveraged a birth cohort with longitudinal biosampling to study *Giardia* infections and their impact on child growth. We found that *Giardia* infection was negatively associated with length-for-age and Insulin-like Growth Factor 1 (IGF-1) levels, positively associated with biomarkers of intestinal epithelial disruption, yet uncoupled from markers of systemic inflammation and intestinal inflammation. The association with poor linear growth, epithelial disruption, and reduced growth signaling from intestinal inflammation may be a unique characteristic of *Giardia* which requires further investigation of its pathophysiology.

## Introduction

*Giardia lamblia* (also known as *Giardia duodenalis* or *Giardia intestinalis*) is a globally distributed intestinal protozoan parasite, primarily transmitted through contaminated water and fecal-oral exposures. An estimated 280 million episodes of symptomatic *Giardia* occur globally each year [1], the majority of which are concentrated in low- and middle-income countries (LMICs). An even greater number of asymptomatic infections occur, yet are infrequently reported. *Giardia* can result in a broad spectrum of clinical outcomes, ranging from asymptomatic carriage to symptomatic diarrhea, conventionally characterized by abdominal cramping, bloating, steatorrhea, and weight loss. Chronic gastrointestinal disturbances also occur [2,3].

In LMICs where *Giardia* is highly endemic, its detection in the stool (regardless of symptoms) has been associated with increased intestinal permeability and reduced anthropometric measurements such as length-for-age [LAZ] and to a lesser extent weight-for-age [WAZ] in colonized children [4]. Notably, the Malnutrition and Enteric Disease study (MAL-ED) identified *Giardia* as one of the top five contributors to linear growth faltering in children under two years of age [5]. Although longitudinal studies have increasingly supported an association between *Giardia* infection and impaired linear growth [5–10], the magnitude of its impact on child growth remains inconsistent across different geographic regions and populations. This variability persists even in multi-site studies following an identical study protocol, suggesting that study design alone does not account for these differences [11]. Further research is needed to investigate host and environmental factors, as well as the role of persistent and recurrent *Giardia* infections, to better understand their contribution to individual-level variations in child growth and development.

The pathogenesis underlying growth failure following *Giardia* infection remains poorly understood. Different potential mechanistic pathways have been described [12]. Evidence suggests that linear growth faltering and intestinal barrier dysfunction may be independent of gut inflammation, as measured by fecal biomarkers commonly associated with Environmental Enteric Dysfunction (EED), such as neopterin and myeloperoxidase [11]. Multiple studies have reported an absence of conventional intestinal markers of inflammation in children with concurrent *Giardia* detection. [4,13,14], yet simultaneously findings of increased intestinal permeability and disruption of nutrient-metabolic pathways [4,11]. Experimental models in gnotobiotic mice have recently demonstrated *Giardia-*mediated impaired growth and inadequate nutrient absorption in the absence of prototypic EED-like chronic lymphocytic inflammation [11,12]. Further research is required to elucidate the precise mechanisms by which *Giardia* contributes to the growth impairment.

Among 76 Nicaraguan children followed from birth through 36 months of age, we captured high-resolution monthly longitudinal growth data, surveillance stool sampling, episodic diarrhea samples, and 49 serum samples to determine the contributions of *Giardia* on linear growth trajectories. Additionally, we profiled indicators of systemic and intestinal inflammation and gut epithelial damage, by testing for associations with asymptomatic infections, and for dose-dependence (e.g., persistent or recurrent *Giardia* infections) with those outcomes.

## Methods

### Ethics statement

The study was approved by the Ethical Committee for Biomedical Research (CEIB) “Dr. Uriel Guevara Guerrero” of the National Autonomous University of Nicaragua (UNAN) at León (Acta number 2–2017; FWA00004523/ IRB00003342) and the Institutional Review Board of the University of North Carolina at Chapel Hill (study number 16– 2079). Written informed consent was obtained from a parent or legal guardian of each participant prior to enrollment in the study. In addition, consent for sample storage and future unspecified analyses was obtained from the caregivers on behalf of their children.

### Participants

This study included a subset of children enrolled in the Sapovirus-Associated GastroEnteritis (SAGE) birth cohort study in León, Nicaragua [15]. In brief, the participant recruitment in the SAGE cohort occurred between June 12, 2017, and July 31, 2018, and included mothers of live-born singleton infants residing in 14 contiguous health sectors of the Perla María Norori Health District in León. Exclusion criteria were estimated gestational age < 36 weeks, birthweight <2,000 g, known chronic health conditions, plans to relocate during the study period, known immune disorders or receipt of blood transfusion in either the infant or mother within the preceding 9 months, or the presence of another household member already enrolled in the birth cohort. The study population comprised families from diverse socioeconomic backgrounds, including high-income households in the urban center and low-income households in peri-urban neighborhoods of León. During the initial household visit at 10–14 days of birth, fieldworkers collected information on child characteristics (sex, mode of delivery, nutritional status), family characteristics (maternal age, education, and employment of household members), and household characteristics (water sources, sanitation system, floor type), the Global Positioning System (GPS) coordinates were also recorded for spatial *Giardia* density analysis. Subsequently, each child was visited weekly in their households from birth until 36 months of age to surveil for acute gastroenteritis (AGE) episodes (defined as diarrhea and/or vomiting) and collect epidemiological data as we described previously [15,16]. Each month, mothers were asked to provide data regarding breastfeeding information and participant height and weight were also measured along the cohort (S1 Fig).

Available resources for *Giardia* detection and bioassays restricted the subset of children in this study to 76 participants. We therefore first restricted the sample to size to only those children who contributed ≥ 90% of expected surveillance samples (≥27 stools over 36 months of follow-up) (N = 107). Among these 107 children, we randomly selected 76. Baseline demographic and epidemiological characteristics of these 76 children did not differ significantly from those of the remaining cohort (S1 Table).

### Specimen collection

Stool specimens were collected in the household within 2 hours of defecation and transported in a sterile plastic container or in a soiled diaper at 4°C to the Microbiology Department of UNAN-León for analysis. For routine stool samples, monthly samples were collected for each child from birth to 24 months of age, and also at 27, 30, 33, and 36 months of age (S1 Fig). Additionally, stool samples were collected from each of the reported AGE episodes, which were defined as at least three loose stools in a 24-hour period, a change in the consistency of stools (bloody, very loose, or watery), or the presence of vomiting, following at least three symptom-free days [15]. When AGE stools were collected at the same time as the routine sampling, these samples were classified as AGE and excluded from the routine stool category. Both routine and AGE stool samples were stored at -20˚C as raw specimens and as 1:10 aliquots (100 mg/ml) in phosphate-buffered saline (PBS) until processing. Samples were stored under these conditions for approximately 24–36 months prior to biomarker analysis. Blood samples were collected in the household by venipuncture at 24 months and transported at 4°C to the Microbiology Department of UNAN-León within 2 hours, serum was extracted by centrifugation and stored at -80˚C until processing.

### Molecular detection of *Giardia*

To identify *Giardia* using Real-Time PCR, 200μl of the 1/10 stool suspension was used to extract DNA using the QIAamp Fast DNA Stool Mini Kit (Cat No./ID: 51604) and by following the manufacturer’s instructions. Stool suspension was initially treated with acid-washed glass beads (0.5 mm; Sigma) and vortexed for 2–5 min, as described by Stroup SE *et al* [17], to increase cyst lysis and DNA extraction. Real-time PCR was performed to identify *Giardia* in AGE stool samples using the protocol described by Verweij JJ *et al* that target the small subunit ribosomal (SSU) gene (18S- like) from *Giardia* (GenBank accession no. M54878) [18]. In brief, 0.2M of the forward and reverse primers and probe (*Giardia* -80F 5’-GAC GGC TCA GGA CAA CGG TT-3’, *Giardia* -127R 5’-TTG CCA GCG GTG TCC G-3’, *Giardia* -105T FAM-5’-CCC GCG GCG GTC CCT GCT AG-3’) were added to a PCR reaction mix consisting of 3 μL of DNA, 12.5 μL of Bio-Rad iQ Multiplex Powermix (Bio-Rad Laboratories, Hercules, CA, USA) and nuclease-free water to a final volume of 25μl. PCR conditions were 95˚C 10 min and 45 cycles: 95˚C 10 s, 60˚C 1 min (signal reading). Real-time PCR was performed using the Bio-Rad CFX96 Touch Real-Time PCR Detection System. The real-time PCR was considered positive if the cycle threshold (Ct) was of ≤35. Only one sample resulted with a Ct value between 36–45. That child otherwise had recurrent *Giardia* positive results. There was therefore negligible risk for misclassification bias based on the Ct threshold cut-off. To control for false-positive detection, nuclease-free water was included as a negative control during the DNA extraction process and as a no-template control in each real-time PCR run. The positive control was ATCC Quantitative Synthetic DNA from *Giardia lamblia* PRA-3006SD with a Ct of 30 ± 2. Laboratory analyses were conducted blinded to the clinical and epidemiological data.

### Biomarkers for pathways of growth-faltering

#### Intestinal biomarkers.

ELISA for fecal Neopterin (NEO), Myeloperoxidase (MPO), and Regenerating family member 1β (Reg-1β) were performed using surveillance stool samples at 12, 24, and 36 months per child (S1 Fig). Samples from children who reported an episode of diarrhea within the month prior to collection were excluded from the analysis. ELISA kits were purchased from commercial vendors (NEO: GenWay Biotech Inc, San Diego, CA. MPO: Immunodiagnostik AG, Stubenwald-Allee, Bensheim, Germany. Reg-1β: RayBiotech Inc, Norcross, GA) and run per the manufacturer’s directions. Stool samples were diluted 1:100 with assay buffer before use in the NEO ELISA kit, 1:500 for use in the MPO ELISA kit, and 1:1000 for use in the Reg-1β ELISA kit. Final concentrations were obtained using the standard curve method. Data were analyzed both raw data and normalized to fecal total protein measured by Pierce BCA Protein Assay Kits (as a way to account for sample protein degradation) performed according to the manufacturer’s directions.

#### Systemic biomarkers.

Serum samples collected at 24 months of age were tested to determine concentrations of intestinal fatty acid binding protein (I-FABP), soluble CD14 (sCD14), insulin-like growth factor 1 (IGF-1), fibroblast growth factor 21 (FGF21), alpha-1 acid glycoprotein (AGP), C-reactive protein (CRP), soluble transferrin receptor (sTfR), retinol binding protein 4 (RBP4) as measured by the MEEDAT 11-plex ELISA (Q-Plex Human Environmental Enteric Dysfunction 11-plex, Quansys Biosciences, USA) as described by Arndt *et al* [19] (S2 Table). Additionally, serum samples were also analyzed for Anti-Flic IgA using ELISA commercial kit (SunLong Biotech Co. LTD, Hangzhou, China) according to the commercial kit’s instructions.

### Statistical analysis

All statistical analyses were performed using R software (version RStudio 2023.09.0 + 463; R Foundation for Statistical Computing, Vienna, Austria). First, the birth characteristics like sex, mode of delivery, mean birth length and weight, socioeconomic status (SES), and breastfeeding data of the children were described using percentages with standard deviations or medians with interquartile ranges (IQR). SES was assessed using a poverty index (PI) based on the criteria described by Peña *et al.* for the Nicaraguan setting [20]. The PI includes five domains: (i) household’s conditions (ii) sanitation facilities, (iii) education level of household members, (iv) occupation of household members, and (v) household overcrowding; each domain contributes one point when the household does not meet the minimum required conditions. Based on these components, households were categorized as no-poor (PI ≤ 1), poor (PI = 2–3), or extremely poor (PI ≥ 4) established based on non-compliance or lack of access to essential indicators for individuals. SES was classified as “missing” when information required to compute any of its components was unavailable for at least one household member. Then, we calculated the frequency and incidence rate (events/100 child-years) for symptomatic and asymptomatic *Giardia* infection throughout the cohort, as well as for persistent and recurrent infection in infected children. A persistent infection in this cohort was defined as two or more consecutive stools with a *Giardia* positive qPCR result, and a recurrent infection was defined as two or more non-consecutive stools with a positive *Giardia* qPCR. Next, we performed a Kernel Density Estimation (KDE) analysis using ArcGIS Software v10.5 to assess the density of *Giardia* infections within the study area.

We assessed correlates of *Giardia* detection, persistent infection, and recurrent infection using a Pearson’s chi-square test or Fisher’s exact test for cell sizes <5 for categorical variables. Potential correlates of interest identified in prior literature included age, gender, water source, delivery mode, floor type, sanitation type and breastfeeding. Categorical characteristics associated with *Giardia* detection below the P = 0.1 type I error level were evaluated for independent associations with *Giardia* detection using a multivariable Cox proportional hazards model to estimate the adjusted relative hazard and 95% confidence interval (CI) for *Giardia* infection. Potential confounders for exposure-outcome were identified through analysis of directed acyclic graphs (S2 Fig)

Monthly weight and length were used to assess growth velocities for length-for-age (LAZ) and weight-for-age (WAZ) using the WHO growth standards for girls and boys [21]. The effect on the growth velocities was performed at 2 levels. First, the Mann-Whitney U test was used to assess the effect of *Giardia* detection on differences (delta [Δ]) on growth outcomes (ΔLAZ and ΔWAZ) between birth measure and measurements at 24 and 36 months old between any precedent *Giardia-*detected and non-detected children throughout the cohort. Second, we conducted linear regression analyses using generalized estimating equations (GEE) to assess the effect of *Giardia* infection on LAZ and WAZ. As an exploratory analysis, we conducted linear regression to test for associations between systemic and fecal biomarker concentrations with contemporaneous LAZ and WAZ measurements.

Effects were estimated using monthly longitudinal LAZ and WAZ measurements for “any *Giardia*”, “recurrent event”, and “persistent event” (S3 Fig). All models were adjusted for child’s age at infection, birth LAZ and WAZ, baseline LAZ and WAZ (according to the model), mode of delivery, sex, SES, breastfeeding, and episodes of diarrhea during the same period. A *P* value <0.05 was considered statistically significant. Furthermore, fecal biomarker levels at 24 and 36 months, as well as systemic biomarkers at 24 months of age, were analyzed using the Mann–Whitney U test, comparing children who had been infected with *Giardia* by those ages with those who had not. Finally, Spearman’s correlation was used to evaluate the relationship between the number of *Giardia* infections and changes in biomarkers, as well as their impact on child growth, measured by WAZ and LAZ. To further characterize the spectrum of *Giardia* detection in relation to clinical presentation, we included predefined exposure categories based on stool PCR results and reported diarrhea at the time of sampling, including *Giardia* detected in non-diarrheal stools, *Giardia* detected in diarrheal stools, diarrhea without *Giardia* detection, diarrhea of any cause, and any *Giardia*. These categories were used for descriptive and exploratory purposes only, including in correlation analyses, and we emphasize that detection of *Giardia* in diarrheal stools does not imply causality. The interpretation of Spearman’s rho coefficient followed the criteria proposed by Schober *et al*. [22]: where values between 0.00 and 0.10 were considered negligible; from 0.10 to 0.39 as weak; 0.40 to 0.69 as moderate; from 0.70 to 0.89 as strong, from 0.90 to 1.00 as very strong correlations. A P value <0.05 was considered statistically significant. Images were generated using GraphPad Prism V7 (GraphPad Software Inc).

## Results

### Epidemiological characteristics of population

Of the 76 children included in this study, 31 (41%) were female, 36 (47%) born vaginally, 73 (96%) of children received non-exclusive breastfeeding with a median duration of 16.5 (IQR: 5.7, 31.2) months (Table 1). Exclusive breastfeeding was short-lived (median of 3.1 weeks, IQR: 0.5, 5.7). Twenty-eight (38%) of children lived in houses not having basic needs met (poverty score ≥2) (Table 1), where 21 (28%) had no indoor toilet and 20 (26%) had an earthen floor at home. Only 5 (7%) of houses did not have potable water at home.

**Table 1:** Epidemiological characteristics of children in the sub-cohort study (n = 76 children).

Characteristics	n (%) or median (IQR)
*Birth characteristic*	
Sex (%Female)	31 (41)
Mode of delivery (%Vaginal)	36 (47)
Mother’s age at birth (years)	20 (23, 26)
Mean birth weight (in kg)	3.2 (3.0, 3.5)
Mean birth length (in cm)	51.3 (50.3, 52.6)
*Socioeconomic and household conditions*	
SES (% poor or extremely poor) *(2 missing)*^α^	28 (37)
Sanitation type (%Latrine)	21 (28)
Floor-type (%Earthen)	20 (26)
Water resources (%Non-potable at home)	5 (7)
*Maternal education attainment (2 missing)*	
Completed primary education or less	21 (28)
Completed any secondary education	53 (72)
*Breastfeeding*	
Ever Breastfed	73 (96)
Median duration of breastfeeding (in months)	16.5 (5.7, 31.2)

α SES was assessed using a poverty index according to Peña et al [20]. SES: Socioeconomic Status.

### Incidence of *Giardia* infections

**Fig 1:**
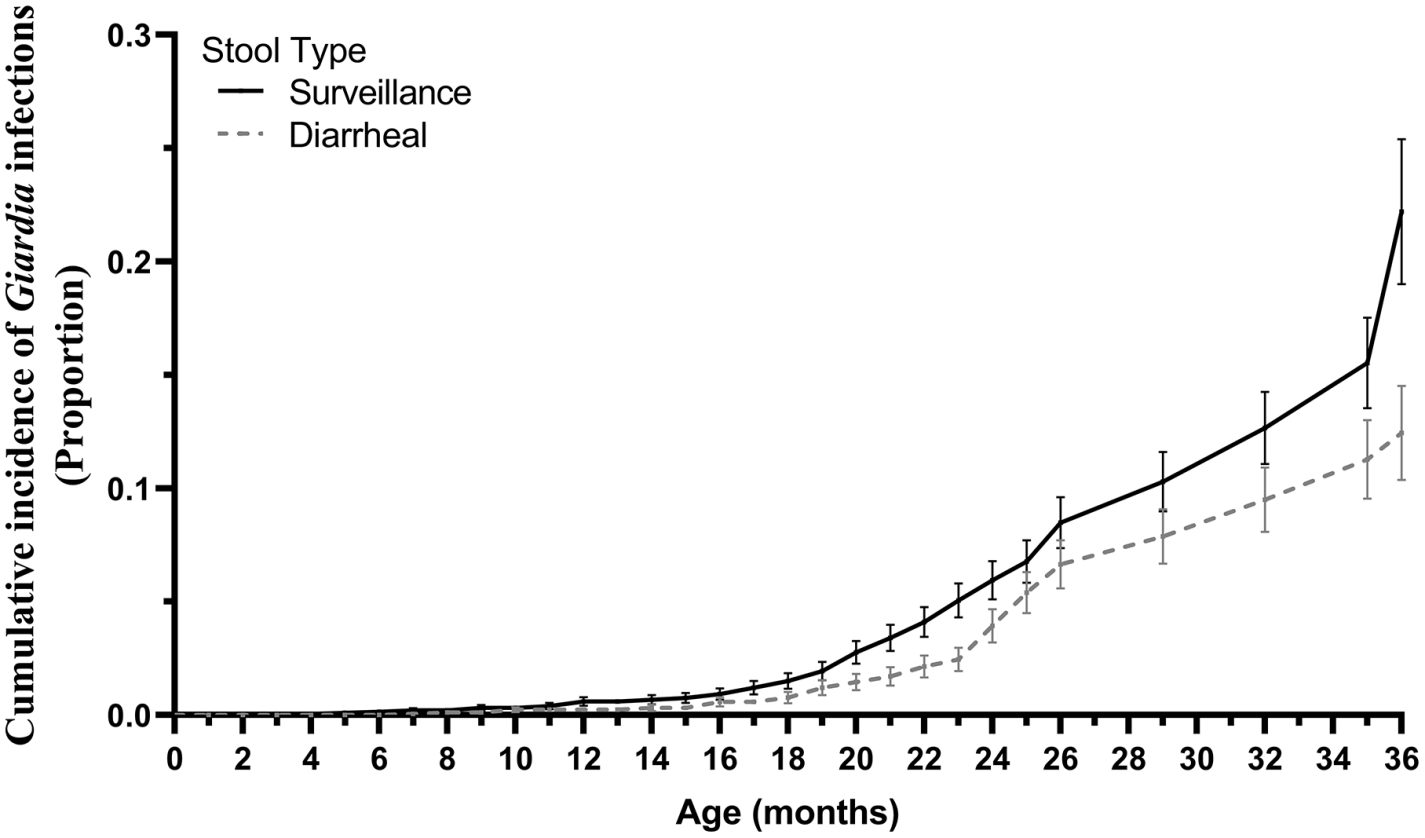
Cumulative incidence of *Giardia* detection in routine (n = 1903) and diarrheal stool (n = 402) samples among the 76 children.

Among the 76 children, 2305 stool samples were collected and tested for *Giardia* by qPCR. 1903 were collected for routine monthly surveillance, and 402 were AGE-stools. A total of 44 of (57.9%) of the 76 children experienced at least one *Giardia* detection. The overall incidence of *Giardia* detections was 59.6 per 100 child-years (95% CI, 49.6–69.7). Incident *Giardia* detection increased from the first year (15.8 [95% CI, 6.8–61.3] per 100 child-years) to the second year (123.7 [95% CI, 98.7–148.7] per 100 child-year) and was 46.1 [95% CI, 30.8–61.3] in the third year (Table 2). Incidence of *Giardia* detection was higher in routine stools compared with diarrheal-stools (35.5 [95% CI, 27.7–43.2] vs 24.7 [95% CI, 18.2–31.3] per 100 child-years) (Table 2 and Fig 1). Throughoutfollow-up, 18 of the 44 children ever infected with *Giardia* (40.9%) had a persistent infection, while 10/44 (22.7%) experienced a reinfection event (Table 2 and S4 Fig). Persistent infections had a median duration of 3 months (IQR: 2–5 months)

**Table 2:** Incidence of *Giardia* detection in routine and diarrheal stools in children from León (n = 76 children).

Events	Incidence^α^
Overall Incidence of *Giardia*-Positive Stool (n = 76)	59.6 (49.6-69.7)
Overall Incidence of *Giardia* in the 1^st^ year^*^	15.8 (6.8-61.3)
Overall Incidence of *Giardia* in the 2^nd^ year^*^	123.7 (98.7-148.7)
Overall Incidence of *Giardia* in the 3^rd^ year^**^	46.1 (30.8-61.3)
Incidence of *Giardia* in routine stools (n = 76)	35.5 (27.7-43.2)
Incidence of *Giardia* in diarrheal stool (n = 74)	24.7 (18.2-31.3)
Incidence of New *Giardia* infections^β^ (n = 76)	34.6 (27.0-42.2)
Incidence of *Giardia* Reinfection Events (n = 44)^⊥^	46.3 (17.6-75.1)
Incidence of Persistent *Giardia* Infections (n = 44)^§^	40.9 (22.1-59.8)

α Incidence per 100 child-years. ^*^Incidence in the 1^st^ and 2^nd^ years was calculated using monthly routine stool samples. ^**^ Incidence in the 3^rd^ year was calculated using quarterly routine stool samples. ^β^New *Giardia* episodes were defined if *Giardia* was detected positive in surveillance or diarrheal stools, and the most recent previous stool tested negative for *Giardia*. ^⊥^*Giardia* re-infection was defined as a child having more than one new *Giardia* episode in non-consecutive stool samples. ^§^Persistent *Giardia* infections were defined as two or more consecutive monthly stool samples that tested positive for *Giardia*.

### Characteristics related to *Giardia* infections

Birth, socioeconomic, and nutritional characteristics were compared between children who had at least one *Giardia* detection (n = 44) and those with no *Giardia* detection during cohort follow-up (n = 32). *Giardia* detections were more frequent among children living in houses not having basic needs met (*P* = 0.013), in houses using latrines for sanitation (as compared to having an indoor toilet) (*P* = 0.012), and those with an earthen floor (*P* = 0.001) (Table 3). The proportion of those socioeconomic indicators was greater among those with >2 detections compared to ≤2 detections (Table 4). Birth characteristics (including sex, mode of delivery, and gestational age), history of an AGE episode of any etiology, and breastfeeding duration were not associated with *Giardia* detection. After adjusting for potential confounders, living without basic needs met (aHR, 2.30 [95% CI, 1.23–4.28]) and having an earthen floor in the home (aHR, 2.90 [95% CI, 1.58–5.50]) remained significantly associated with an increased risk of *Giardia* detection (Table 5).

**Table 3:** Epidemiological characteristics of children with and without *Giardia* infections detected in the first three years of life (n = 76 children).

Characteristics	n (%) or median (IQR)		P value*
	Children with *Giardia* detection (n = 44)	Children without *Giardia* detection (n = 32)	
*Birth characteristic*			
Sex (%Female)	17 (38.6)	14 (43.8)	0.654
Mode of delivery (%Vaginal)	20 (45.5)	16 (50.0)	0.695
Mother’s age at birth (years)	23 (20, 25)	25 (22, 28)	0.066
Mean birthweight (in kg)	3.2 (3.0, 3.5)	3.1 (2.9, 3.5)	0.185
*AGE history*			
Mean of total diarrhea at 3 years	5 (3, 8)	4 (3, 7)	0.722
*Socioeconomic and household conditions*			
**SES (% poor or extremely poor) *(2 missing)*α**	**21 (50)**	**7 (21.9)**	**0.013**
**Sanitation type (%Latrine)**	**17 (38.6)**	**4 (12.5)**	**0.012**
**Floor-type (%Earthen)**	**18 (40.9)**	**2 (6.3)**	**0.001**
Water resources (%Non-potable at home)	4 (9.1)	1 (3.1)	0.300
*Maternal education attainment (2 missing)*			
Completed primary education or less	12 (27.9)	9 (29.0)	0.916
Completed any secondary education	31 (72.1)	22 (71.0)	0.916
*Nutrition*			
Ever Breastfeeding	42 (95.5)	31 (96.9)	0.754
Breastfeeding durations (in months)	13 (4, 32)	21 (8, 31)	0.415
Median WAZ at birth	1.22 (0.53, 1.78)	1.13 (0.39, 1.66)	0.394
Median LAZ at birth	0.65 (0.05, 1.55)	0.71 (0.09, 1.66)	0.712

AGE: acute gastroenteritis. SES: Socioeconomic status. α SES was assessed using a poverty index according to Peña et al [20]. *Pearson’s chi-square test or Fisher’s exact test for cell sizes <5 for categorical variables. Mann-Whitney U test was used for numerical variables.

**Table 4:** Epidemiological characteristics of children with less or equal and more than 2 of *Giardia* infections detected during the first three years of life, compared to children without *Giardia* infection.

Epidemiological characteristics	Children without *Giardia* detection (n = 32)* Ref.	Children with ≤2 *Giardia* infections (n = 23)*	P. value^⊥^	Children with >2 *Giardia* infections (n = 21)*	P. value^⊥^
*Birth characteristic*					
Sex (%Female)	14 (43.8)	8 (34.8)	0.503	9 (42.9)	0.949
Mode of delivery (%Vaginal)	16 (50.0)	12 (52.2)	0.874	8 (38.1)	0.394
Mother’s age at birth (years)	25 (22, 28)	23 (19, 25)	0.240	23 (20, 26)	0.569
Mean birthweight (in kg)	3.1 (2.9, 3.5)	3.3 (3.0, 3.7)	0.261	3.2 (3.0, 3.6)	0.501
*AGE history*					
Median of total diarrhea at 3 years	4 (3, 7)	6 (4, 8)	0.070	5 (2, 7)	0.763
*Socioeconomic and household conditions*					
**SES (% poor or extremely poor) *(2 missing)***^**α**^	7 (21.9)	9 (40.9)	0.132	**12 (60.0)**	**0.005**
**Sanitation type (%Latrine)**	4 (12.5)	**9 (39.1)**	**0.022**	**8 (38.1)**	**0.029**
**Floor-type (%Earthen)**	2 (6.3)	**8 (34.8)**	**0.007**	**10 (47.6)**	**<0.001**
Water resources (%Potable at home)	31 (96.9)	19 (82.6)	0.069	21 (100)	0.413
Maternal education attainment *(2 missing)*					
Completed primary education or less	9 (29.0)	4 (17.4)	0.322	8 (40.0)	0.417
Completed any secondary education	22 (71.0)	19 (82.6)	0.322	12 (60.0)	0.417
*Nutrition*					
Ever Breastfeeding	31 (96.9)	22 (95.7)	0.811	20 (95.2)	0.760
Breastfeeding duration (in months)	21 (8, 31)	8 (4, 28)	0.109	24 (5, 35)	0.942
Mean WAZ at birth	1.13 (0.39, 1.67)	1.51 (0.85, 1.87)	0.116	0.99 (0.19, 1.58)	0.841
Mean LAZ at birth	0.71 (0.09, 1.66)	0.65 (-0.12, 1.51)	0.804	0.65 (0.14, 1.67)	0.709

Ref: Reference group. *n (%) or median (IQR). ^⊥^Compared to children without *Giardia*. NA: not applicable. AGE: acute gastroenteritis. ^α^ SES was assessed using a poverty index according to Peña *et al* [20]. ^⊥^Pearson’s chi-square test or Fisher’s exact test for cell sizes <5 for categorical variables. Mann-Whitney U test was used for numerical variables.

**Table 5:** Association between selected characteristics and *Giardia* detection in a birth cohort study (n = 76 children).

Characteristic^§^	Crude		Adjusted*	
	HR	95% CI	HR	95% CI
**SES (% poor or extremely poor) *(2 missing)*α**	**2.31**	**1.26, 4.25**	**2.30**	**1.23, 4.28**
Sanitation type (%Latrine)	2.35	1.28, 4.33	1.35	0.62, 2.95
**Floor-type (%Earthen)**	**2.90**	**1.58, 5.33**	**2.51**	**1.15, 5.50**

§Model adjusted was created using all categorical variables with a P < 0.1 in bivariate predictors from Tables 3 and 4. HR: Hazard Ratio. CI: Confidence Interval. αSES was assessed using a poverty index according to Peña *et al* [20].

*Adjusted analyses were not performed for non-significant variables. Adjusted models included potential confounders for each variable identified through analysis of causal diagrams. SES was adjusted for gestational age, water source, and delivery mode; floor type was adjusted for water source and sanitation type; sanitation type was adjusted for floor type and water source.

### Seasonality and geospatial distribution

*Giardia* was detected across all months of the year (S5A Fig), with no statistically significant difference between the prevalence of 7.6% in the dry season (November-April) vs 5.23% in the rainy season (May – October) (P = 0.101; S5B Fig). Geospatially, *Giardia* was detected throughout the area studied (S5C Fig), and the Kernel Density Estimation (KDE) analysis performed demonstrates a single zone of high density of *Giardia* infections, with an estimated value of 22 events per km² (S5D Fig) in 10/15 children living inside this area, in which the use of latrine (P = 0.013) and have a poor or extremely poor SES (P = 0.023) were higher comparing with children living outside of the high area burden (S3 Table). This indicates a marked concentration of events in this specific area, with no other significant density clusters identified across the study region.

### Effects on growth

**Fig 2:**
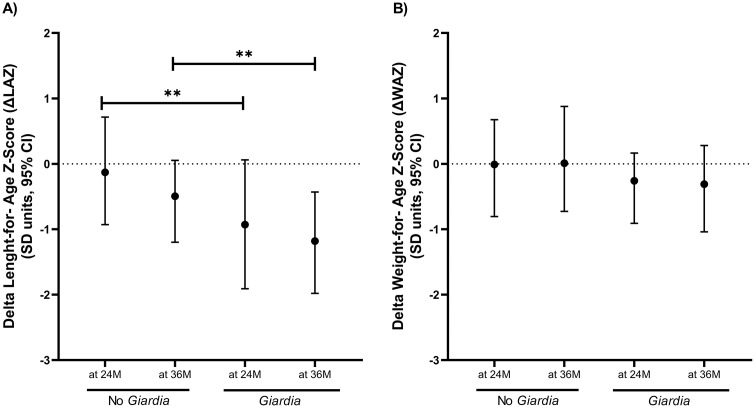
Difference in Length-for-age (ΔLAZ) and weight-for-age (ΔWAZ) Z-score between birth and measurements at 24 and 36 months of age. Panel (A) shows ΔLAZ, and panel (B) shows ΔWAZ in children who experienced at least once *Giardia* detection by 24 months (24M) (*Giardia*, n = 35) and 36 (36M) months of age (n = 44), and children without *Giardia* detection at 24M (No *Giardia*, n = 41) and 36M (n = 32). *P < 0.050 to 0.010, **P < 0.010 to 0.001, ***P < 0.001.

**Fig 3:**
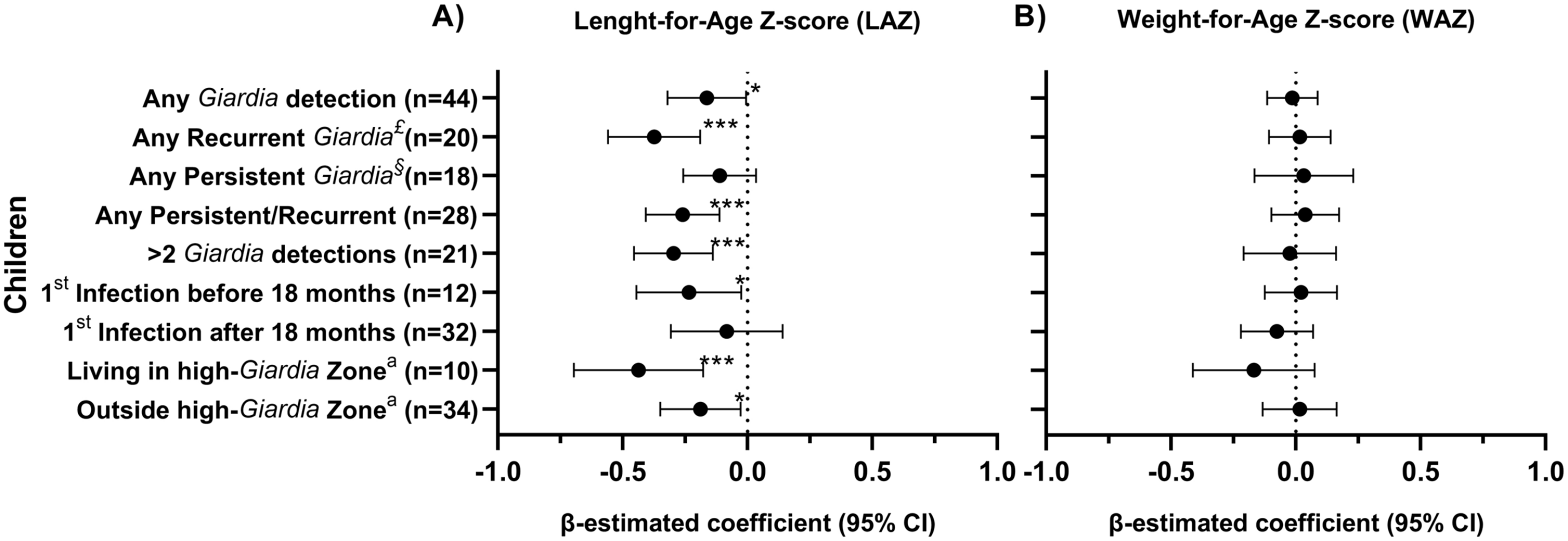
Estimation of linear regression in children infected with *Giardia* (n = 44). A) Length-for-age (LAZ) and B) Weight-for-age (WAZ) Z-score in infected children. The β-estimated coefficient was calculated using generalized estimating equations (GEE), and the value one month prior to the *Giardia* infection was used as the baseline. Models were adjusted for age, mode of delivery, sex, SES, birth WAZ and LAZ, baseline WAZ or LAZ (S3 Fig), breastfeeding, and episodes of diarrhea. ^£^Any recurrent infection was defined if a child had more than one *Giardia*-positive qPCR result in non-consecutive stool samples. ^§^Any persistent *Giardia* infections were defined if two or more consecutive routine stool samples that tested positive for *Giardia*. ^a^Children living within or outside high-*Giardia* burden areas based on the Kernel density distribution of *Giardia* infections. *P < 0.050 to 0.010, **P < 0.010 to 0.001, ***P < 0.001.

Growth outcomes were first evaluated using the difference (Δ) between birth and anthropometric measurements (ΔLAZ and ΔWAZ) at 24 and 36 months of age in children with at least one *Giardia* detection compared to those without any detection during the cohort. Children with *Giardia* detection showed significantly lower ΔLAZ at 24 (median: -0.93 vs -0.13, P = 0.006) and 36 months (median: -1.18 vs -0.50, P = 0.007) (Fig 2A). No significant differences were observed in ΔWAZ at 24 (median: -0.26 vs -0.01, P = 0.253) or 36 months of aged (median: -0.31 vs 0.01, P = 0.105) (Fig 2B). Regression models adjusted for age, mode of delivery, sex, socioeconomic status, breastfeeding, and episodes of diarrhea among the 44 *Giardia*-infected children (S4 Table) estimated that *Giardia* detection was associated with reduced LAZ at 36 months among children with: any *Giardia* detection (β:-0.16, 95% CI: -0.01, -0.32, P = 0.042) (Fig 3A), any persistence or recurrent infections (β:-0.26, 95% CI: -0.11, -0.41, *P* = < 0.001), and more than two *Giardia* detections during the surveillance period (β:-0.29, 95% CI: -0.14, -0.45, *P* = < 0.001) (Fig 3A). Using a data-driven categorization of age at first *Giardia* infection (0–18 and 18–36 months), we observed a negative association with LAZ among children infected within the first 18 months of life (β:-0.24, 95% CI: -0.03, -0.45, *P* = 0.028) (Fig 3A and S4 Table), whereas a numerical decrease in LAZ among children infected after 18 months of age did not reach significance. The geospatial distribution of *Giardia* density was also associated with LAZ. Although reductions in LAZ were observed in children both inside and outside high-density areas, the effect was greater among those living in high-density areas, with a reduction of 0.44 units in LAZ (β:-0.44, 95% CI: -0.18, -0.69, *P* = < 0.001), as compared to those living in lower density areas, who had a reduction of -0.19 units (β:-0.19, 95% CI: -0.03, -0.35, *P* = 0.022) (Fig 3A). No differences were observed in WAZ (Fig 3B).

### *Giardia* does not associate with sustained increases in fecal biomarkers of EED

**Fig 4:**
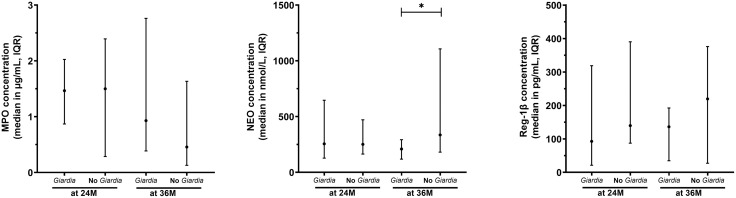
Fecal biomarkers measured in children with and without *Giardia* infections. Fecal biomarkers measured at 24 (24M) and 36 months of age (36M) in children infected at least once with *Giardia* infections at 24M (*Giardia*, n = 24) and 36M (n = 31), and children not infected at 24M (No *Giardia*, n = 34) and 36M (n = 27) for: A) Myeloperoxidase (MPO), B) Neopterin (NEO), C) Regenerating family member 1β (Reg-1β). *P < 0.050 to 0.010, **P < 0.010 to 0.001, ***P < 0.001.

Fecal NEO, MPO, and Reg-1β concentrations were analyzed in the 58 children who had samples available at both 24 and 36 months of age (Fig 4 and S5 Table). All fecal biomarker concentrations were non-normally distributed at both time points. Total fecal protein was also quantified per sample and used to normalize biomarker concentrations (S6 Fig). Fecal biomarkers were compared between children with any prior *Giardia* detection (24/58 at 24 months and 31/58 at 36 months) and those without *Giardia* detection (Fig 4A-4C). Only mean NEO at age 36 months was significantly lower (209.1 vs 335.4 nmol/L, *P* = 0.024) in children with *Giardia* infections (Fig 4B).

### Association between *Giardia* infection and systemic biomarkers

**Fig 5:**
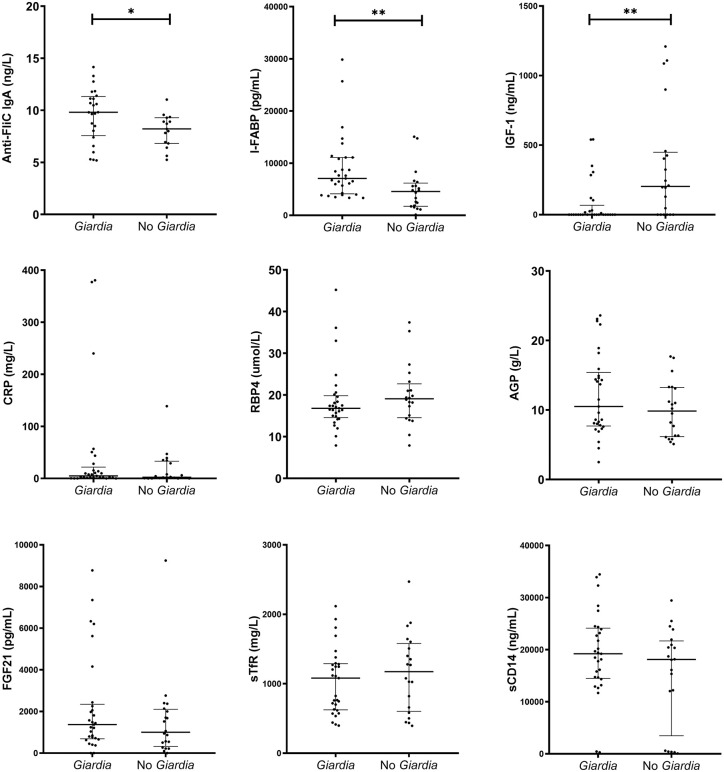
Systemic biomarkers measured at 24 months of age in children infected at least once with *Giardia* infections (*Giardia*, n = 29) and children not infected (No *Giardia*, n = 20). A) Anti-flagellin C (Anti-FliC) -IgA. B) Insulin-like growth factor-1 (IGF-1). C) Intestinal fatty acid binding protein (I-FABP). D) Fibroblast growth factor 21 (FGF21). E) Soluble transferrin receptor (sTfR). F) C-reactive protein (CRP). G) Retinol binding protein 4 (RBP4). H) α-1-acid glycoprotein (AGP). I) Soluble cluster of differentiation 14 (sCD14). *P < 0.050 to 0.010, **P < 0.010 to 0.001, ***P < 0.001.

Systemic biomarkers were evaluated at 24 months of age, and compared between children who had at least one *Giardia* detection regardless of symptoms (n = 29) and those without any *Giardia* detection (n = 20) (Fig 5 and S6 Table). The systemic biomarker profiles of children with *Giardia* detection during the first 24 months were characterized by higher concentrations of Anti-FliC IgA (9.8 [IQR: 7.9-11.2] vs 8.2 [IQR: 6.9-9.1] ng/L, *P* = 0.033) (Fig 5A), and I-FABP (7,074.8 [IQR: 4,271.4-11,085.8] vs 4,573 [IQR: 1,807.4-5,823.7] pg/mL, *P* = 0.002) (Fig 5B), and lower concentrations of IGF-1 (0.1 [IQR: 0.1-31.3] vs 203.3 [IQR: 0.09-433.6] ng/mL, *P* = 0.005) (Fig 5C), compared to children without *Giardia* detection. Both IGF-1 and I-FABP were associated with LAZ at 24 months of age. No significant associations were observed for CRP, RBP4, AGP, FGF21, sTfR, and sCD14 (Fig 5D-5I).

In linear regression models, independent of *Giardia* infection, for every doubling of IGF-1, there was an average of 0.07 standard deviation increase in LAZ (95% CI: 0.005-0.136, p = 0.036). Conversely, for every doubling of AGP, an average of -0.694 standard deviation decrease in LAZ (95% CI: -1.250 to -0.134, p = 0.016) and every doubling of I-FABP, an average of -0.273 standard deviation decrease in LAZ (95% CI: -0.538 to -0.008, p = 0.044) (S7 Table).

### Outcomes correlate with *Giardia* infection

**Fig 6:**
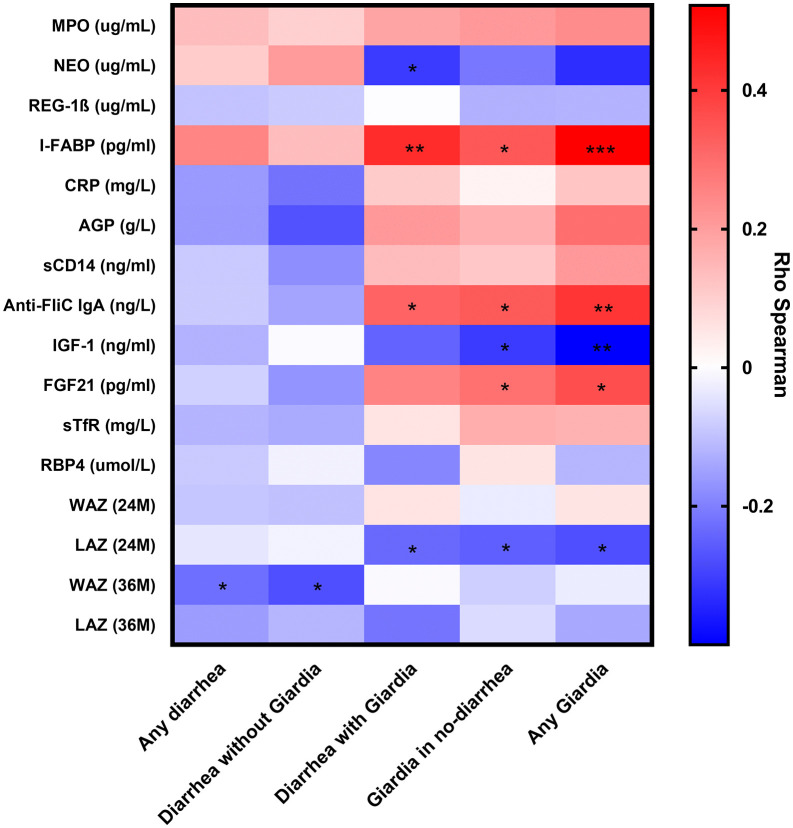
Correlation between fecal and systemic biomarkers with total events of any diarrhea, diarrhea with a without *Giardia*, *Giardia* in no diarrhea stools, and any *Giardia* detection. Correlations were performed using Spearman’s rank correlation with all episodes or infections reported in 49 children throughout the cohort for anti-flagellin C (Anti-FliC)-IgA, insulin-like growth factor-1 (IGF-1), intestinal fatty acid binding protein (I-FABP), fibroblast growth factor 21 (FGF21), soluble transferrin receptor (sTfR), C-reactive protein (CRP), retinol binding protein 4 (RBP4), α-1-acid glycoprotein (AGP), Length-for-age (LAZ), and Weight-for-age (WAZ) Z-score. *P < 0.050 to 0.010, **P < 0.010 to 0.001, ***P < 0.001.

We evaluated the correlation between the number of any diarrhea event, diarrhea with and without *Giardia* detection, *Giardia* in non-diarrhea stool, and any *Giardia* throughout the entire study period with the anthropometric measurements and biomarkers (Fig 6). This approach allowed comparison of potential differential effects with and without diarrhea, rather than implying causality. When analyzing the number of any diarrhea, no significant correlation was found between biomarkers and anthropometric measurements, except for WAZ at 36 months, which showed a weak negative correlation (rho: -0.226, P = 0.049), indicating that for increasing diarrheal episodes by any cause, WAZ tends to decrease slightly. In contrast, when increasing number of diarrhea with *Giardia* detection, a moderate positive corretation was observed for levels of I-FABP (rho: 0.462, *P* < 0.001) and Anti-FliC IgA (rho: 0.311, *P* = 0.043) levels, and weak negative correlation with LAZ at 24 months (rho: -0.236, *P* = 0.040) (Fig 6). Correlations were also assessed using the total number of any *Giardia* detections, showing a moderate negative correlation with IGF-1 (rho: -0.433, *P* = 0.002), and LAZ at 24 months (rho: -0.277, *P* = 0.015), and positive correlations with I-FABP (rho: 0.506, *P* < 0.001), Anti-FliC IgA (rho: 0.412, *P* = 0.008), and FGF21 (rho: 0.362, *P* = 0.011). Similar findings were found when restricting the analysis to non-diarrheal surveillance stools when *Giardia* was detected, suggesting that *Giardia*, rather than stool consistency, accounted for these observations.

## Discussion

We leveraged a birth cohort study from León, Nicaragua [15] to study the natural history of *Giardia* infections and their impact on child health. This birth cohort provides data to study early-life *Giardia* infections, including epidemiology, growth measurements, and fecal and systemic biomarkers. We previously reported that *Giardia* was found in 7.5% of AGE episodes from the entire cohort data [16]. In this study, we focused our examination on the subcohort of children who provided complete monthly surveillance data (stool samples and demographics) to estimate the longitudinal effect of *Giardia* on growth outcomes and biomarkers of intestinal responses. By the end of the 36-month surveillance period, we found that 57.9% of children had at least one *Giardia* infection, with acquisitions beginning around 12 months of age, and incidence progressively increasing through the second year of life. This pattern is consistent with previous findings from León, Nicaragua, where *Giardia* infections tend to occur at older than 24 months of age in this setting [23]. Consistent with findings in the multi-site MAL-ED study, most of the first *Giardia* detections occurred in surveillance stools rather than in diarrheal stools, and persistent and repeated infections were common.

Consistent with previous reports linking *Giardia* to poverty and inadequate sanitation systems [24,25], our findings indicate that household-level deprivations, specifically the absence of basic needs and the presence of earthen floors, are strongly associated with an increased probability of *Giardia* infection, independent of seasonality, with this relationship being more pronounced among children with more than two detections. These results are similar to data from this cohort reported previously, where AGE with *Giardia* detection (AGE-G) was associated with living in a household with a latrine and earthen floor [16], supporting that household environmental conditions may be an important determinant of the burden of exposure to the parasite. Disentangling the independent effects of *Giardia* infection and socioeconomic conditions on child growth remains challenging. Although our models adjusted for key socioeconomic indicators, including a composite poverty index and household characteristics, residual confounding is likely given the close relationship between enteric infections and living conditions. Therefore, the observed association between *Giardia* infection and reduced LAZ may reflect both direct effects of infection and indirect effects related to adverse environmental exposures. These findings are consistent with the possibility that underlying dietary and intestinal microbiota factors mediate *Giardia* outcomes that have been suggested elsewhere [11].

We detected *Giardia* during all months of the year, including during both rainy and dry seasons. Our findings suggest that *Giardia* infections were more prevalent in a specific geospatial area. We also identified a high-density area of *Giardia* infections characterized by low socioeconomic status and predominant use of latrines, which may contribute to the increased burden of *Giardia* in this area. Although further analysis could be conducted to explore the influence of different bandwidth settings on the density distribution, our results are in line with the finding that the geospatial distribution of the intestinal parasitic infections is influenced by socioeconomic conditions [25].

These findings highlight the relevance of geospatial and neighborhood contexts for both the frequency and incidence of infection, and point to potential opportunities for interventions that could reduce the burden of *Giardia* in this area and limit its spread to surrounding communities, as such community-level measures are likely to achieve larger and more durable reductions in transmission than isolated, household-level efforts alone. At the community level, these include health education, promotion of personal hygiene practices, and improvements in sanitation to reduce exposure to [26–28].

Our results show a significant *Giardia-*associated decrease of LAZ at 24 and 36 months of age, regardless of symptoms (e.g., stool consistency). There was a greater, nearly two-fold, impact in those children in whom *Giardia* was detected more than 2 times, including those with persistent or recurrent infections. Our findings are consistent with other studies in LMICs, which have found that *Giardia* is negatively associated with childhood linear growth [5,6,8–10]. Children infected within the first 18 months of life showed a negative association with linear growth, whereas no such effect was observed among those whose first infection occurred after 18 months. These findings support the hypothesis that earlier exposure may have a greater impact on linear growth. On the other hand, neopterin decreased significantly after *Giardia* infection. Similar findings were observed when comparing children who were infected with *Giardia* vs those without *Giardia*. Our study adds to emerging and consistent findings that *Giardia* is unlikely to produce intestinal inflammation during infection [4,13,14], despite associating with impaired linear growth. These findings suggest that impaired linear growth associated with *Giardia* infections appears to be a process independent of typical EED-like inflammation, and instead may be due to the direct disruptions in epithelial cells by parasite factors [29], including secreted proteins and the unique metabolic properties of the protozoan, and/or an interaction with resident intestinal microbes or other gut pathogens [12].

Interestingly, although there was no evidence of typical intestinal inflammation, systemic biomarkers like I-FABP and anti-FliC IgA were increased in children with at least one *Giardia* detection, regardless of the symptoms. These findings are consistent with separately reported associations of increased I-FABP [30] and anti-FliC IgA [6] levels in children following *Giardia* infection, supporting that *Giardia* is associated with intestinal epithelial disruption through mechanisms independent of conventional fecal EED biomarkers. On the other hand, IGF-1 was decreased in *Giardia*-infected children. To our knowledge, this is the first study to suggest that *Giardia* is associated with decreased systemic IGF-1 concentration. While IGF-1 is a well-recognized hormonal driver of linear growth, its relationship with *Giardia* infection has not been previously explored. This evidence suggests that *Giardia* may influence growth outcomes through pathways involving nutrient-metabolic disruption and endocrine modulation. A conceptual model suggests that malnutrition inhibits hepatic IGF-1 synthesis [31], this hypothesis was supported by mouse models in which IGF-1 levels are reduced during malnutrition [32].

Deprivation of amino acids has been also described as one of the different proposed mechanisms that could be associated with the effect on growth as part of the nutrient-metabolite disruption during *Giardia* infections [12]. Both essential and non-essential ammio acids have been reported to be decreased in some children infected with *Giardia* who concomitantly demonstrate poor growth [11]. This may arise through shortening of the intestinal brush-border microvilli, which could lead to lose the intestinal absorptive function [11,33–36], or altered amino acid availability due to parasite metabolism and/or interactions with intestinal microbiota. Amino acid deprivation has been shown to decrease IGF-1 mRNA expression in hepatocytes and muscle cells [37]. Additionally, certain amino acids, such as arginine, can specifically stimulate IGF-1 secretion and act through both growth hormone-dependent and independent pathways to regulate IGF-1 secretion [38].

This observational study has some limitations. First, available resources were sufficient to include a maximum of 76 children from the SAGE cohort. Although a prior power calculation was not performed, this sample size was sufficient to detect effects in our outcomes, including those related to *Giardia* infection. We acknowledge that the sample size limits statistical power to detect smaller effects. However, with 76 cases and an incidence rate of 59.6/100 child-years, we had sufficient power to estimate a CI of width 27 at alpha = 0.05 compared to our CI of width 20, suggesting that our study was slightly underpowered. Second, during the third year of follow-up, stool sampling was performed quarterly rather than monthly, potentially reducing the sensitivity to detect incident infections. Third, detailed nutritional data were not collected in the cohort, including information on complementary feeding (food groups, and quantity), as well as food availability, access, consumption, and nutrient absorption. Additionally, the effects of specific pathogens were assessed only in the context of diarrheal episodes of any cause; specific pathogens were not included as covariates in the analysis. We also acknowledge the lack of adjustment for multiple comparisons (e.g., Benjamini–Hochberg test for false discovery rate), which may increase the likelihood of type I errors. An additional limitation is that on-site sample handling including stool samples that were diluted and stored at −20 °C for an extended period (24–36 months) prior to biomarker analysis. This storage condition may be suboptimal for long-term preservation and could potentially affect biomarker stability (even despite our attempt to account for this using normalizing to total protein). Finally, systemic biomarker measurements were only available at the 2-year time point. Longitudinal data beyond two years would provide a better understanding of the durable impact of *Giardia* infection during early childhood. Similarly, to obtain a comprehensive view of the endocrine axis, metabolic pathways, including growth hormone and insulin-like growth factor binding proteins, should be assessed.

In summary, our study provides an update of the natural history of *Giardia* infection in early childhood, to better understand timing of acquisition, association with symptoms, risk factors, geographical distribution, and its impact on child growth and EED. Our study also provided evidence that *Giardia* infection is negatively associated with LAZ, markers of chronic intestinal damage, and IGF-1 and positively associated with markers of intestinal epithelial disruption. The uncoupling of poor linear growth, epithelial disruption, and reduced growth signaling from intestinal inflammation seen in this study, other cohorts, and experimental models of giardiasis, is supporting a unique characteristic of endemic pediatric giardiasis that requires further investigation of its pathophysiology to guide future interventions.

## Supporting information

S1 Table Baseline epidemiological characteristics of the sub-cohort versus the remaining cohort. (DOCX)

S2 Table Biomarkers Associated with Growth, Systemic Inflammation, and Nutritional Status. (DOCX)

S3 Table Epidemiological characteristics of children living inside vs outside Giardia burden area (n = 76 children). (DOCX)

S4 Table β-estimated coefficient of linear regression using GEE on child anthropometric indicators in children infected with *Giardia* (n = 44). (DOCX)

S5 Table Fecal biomarkers measured at 24 and 36 months of age in children infected at least once with *Giardia* infections and children not infected. (DOCX)

S6 Table Systemic biomarkers measured at 24 months of age in children infected at least once with *Giardia* infections (*Giardia*, n = 29) and children not infected (No *Giardia*, n = 20). (DOCX)

S7 Table β-estimates from linear regression models of log2-transformed fecal and systemic biomarker concentrations on child anthropometric indicators at 24 months of age. (DOCX)

S1 Fig Timeline of routine stool collections, anthropometric assessment (weight-for-age [WAZ] and length-for-age [LAZ]), and timepoints of fecal and systemic biomarker measurements. (TIF)

S2 Fig Causal diagram used for adjusting for potential confounders. Yellow circles with a triangular bullet (‣) represent exposure variables. The red circle represents an ancestor of exposure and outcome. Blue circles with a blue border represent an ancestor of the outcome. Yellow circle with a centrally positioned uppercase letter “I” represents the outcome. The green line means causal path, and the red line means bias pathway.
(TIF)

S3 Fig Diagram illustrating the linear regression models using generalized estimating equations (GEE) to assess the effect of *Giardia* infection on Length-for-Age (LAZ) and Weight-for-Age (WAZ) Z score. Effects were estimated using monthly longitudinal LAZ and WAZ measurements. The model for any *Giardia* infection began at the first positive *Giardia* infection (red circle “b”), using the value one month prior as the baseline (green triangle “a”). The recurrent event models began at the second non-consecutive *Giardia* positive stool (red circle “c”), with the baseline defined as one month prior to this event (green triangle). The persistent event model started at the second consecutive positive stool (red circle “d”), with the baseline defined as one month prior to this event (green triangle). All models were adjusted for child’s age, mode of delivery, sex, socioeconomic status, breastfeeding, and episodes of diarrhea during the same period. *A *P* value <0.05 was considered statistically significant.
(TIF)

S4 Fig Timing of *Giardia* infections in surveillance and diarrheal stools among 76 children. The Y-axis represents child ID, while the X-axis represents the child’s age in months. *Giardia* detection in stools samples was determined using qPCR. Light gray squares: samples with no *Giardia* detection. Yellow squares: surveillance stool samples with a positive *Giardia* detection. Red squares: diarrheal stool samples with positive *Giardia* detection. White squares: monthly stools samples that were not collected. Dark gray squares: diarrheal stools with a *Giardia* negative detection.
(TIF)

S5 Fig Distribution of *Giardia* infections in 76 children during the first 3 years of life. A) Monthly absolute distribution from June 2017 to March 2021. B) Frequency of *Giardia* positive stools by season, rainy (from March to September) and dry season (from November to April). C) Spatial distribution of 76 children included in the study, red points represent children infected with *Giardia* (n = 44). D) Kernel Density Distribution of *Giardia* infections. Darker area represents geographic zone (km^2^) with higher concentrations of infection events. *The base layer of the maps was derived from OpenStreetMap (OpenStreetMap contributors;* *https://www.openstreetmap.org), which is made available under the Open Database License (ODbL;* *https://www.openstreetmap.org/copyright). The maps were created using ArcGIS software (Esri) under an institutional license provided by the University of North Carolina at Chapel Hill. All other elements of the map are original to the authors.*
(TIF)

S6 Fig Fecal biomarkers measured at 24 and 36 months of age in children infected at least once with *Giardia* infections at 24 (*Giardia*, n = 24) and 36 months of age old (n = 31), and children not infected at 24M (No *Giardia*, n = 34) and 36M (n = 27) normalized to total fecal proteins (A) for: B) Neopterin (NEO). C) Myeloperoxidase (MPO), and D) Regenerating family member 1β (Reg-1β). *P < 0.050 to 0.010, **P < 0.010 to 0.001, ***P < 0.001.
(TIF)

## References

[1] Esch KJ, Petersen CA. Transmission and epidemiology of zoonotic protozoal diseases of companion animals. Clin Microbiol Rev. 2013;26(1):58–85. doi: 10.1128/CMR.00067-12 23297259 PMC3553666

[2] Painter J, Gargano J, Collier SA, Yoder JS. Giardiasis Surveillance — United States, 2011–2012. MMWR Surveill Summ. 2015;64:15–25. 25928582

[3] Certad G, Viscogliosi E, Chabé M, Cacciò SM. Pathogenic mechanisms of Cryptosporidium and Giardia. Trends in Parasitology. 2017. doi: 10.1016/j.pt.2017.02.006 28336217

[4] Rogawski ET, Bartelt LA, Platts-Mills JA, Seidman JC, Samie A, Havt A, et al. Determinants and Impact of Giardia Infection in the First 2 Years of Life in the MAL-ED Birth Cohort. J Pediatric Infect Dis Soc. 2017;6(2):153–60. doi: 10.1093/jpids/piw082 28204556 PMC5907871

[5] Rogawski ET, Liu J, Platts-Mills JA, Kabir F, Lertsethtakarn P, Siguas M, et al. Use of quantitative molecular diagnostic methods to investigate the effect of enteropathogen infections on linear growth in children in low-resource settings: longitudinal analysis of results from the MAL-ED cohort study. Lancet Glob Health. 2018;6(12):e1319–28. doi: 10.1016/S2214-109X(18)30351-6 30287125 PMC6227248

[6] Iqbal NT, Syed S, Kabir F, Jamil Z, Akhund T, Qureshi S, et al. Pathobiome driven gut inflammation in Pakistani children with Environmental Enteric Dysfunction. PLoS One. 2019;14(8):e0221095. doi: 10.1371/journal.pone.0221095 31442248 PMC6707605

[7] Fauziah N, Aviani JK, Agrianfanny YN, Fatimah SN. Intestinal Parasitic Infection and Nutritional Status in Children under Five Years Old: A Systematic Review. Trop Med Infect Dis. 2022;7(11):371. doi: 10.3390/tropicalmed7110371 36422922 PMC9697828

[8] Sackey M-E, Weigel MM, Armijos RX. Predictors and nutritional consequences of intestinal parasitic infections in rural Ecuadorian children. J Trop Pediatr. 2003;49(1):17–23. doi: 10.1093/tropej/49.1.17 12630715

[9] Carvalho-Costa FA, Gonçalves AQ, Lassance SL, Silva Neto LMd, Salmazo CAA, Bóia MN. Giardia lamblia and other intestinal parasitic infections and their relationships with nutritional status in children in Brazilian Amazon. Rev Inst Med Trop Sao Paulo. 2007;49:147–53. doi: 10.1590/S0036-46652007000300003 17625691

[10] Das R, Palit P, Haque MA, Levine MM, Kotloff KL, Nasrin D, et al. Symptomatic and asymptomatic enteric protozoan parasitic infection and their association with subsequent growth parameters in under five children in South Asia and sub-Saharan Africa. PLoS Negl Trop Dis. 2023;17(10):e0011687. doi: 10.1371/journal.pntd.0011687 37816031 PMC10588856

[11] Giallourou N, Arnold J, McQuade ETR, Awoniyi M, Becket RVT, Walsh K, et al. Giardia hinders growth by disrupting nutrient metabolism independent of inflammatory enteropathy. Nat Commun. 2023;14(1):2840. doi: 10.1038/s41467-023-38363-2 37202423 PMC10195804

[12] Gutiérrez L, Bartelt L. Current Understanding of Giardia lamblia and Pathogenesis of Stunting and Cognitive Deficits in Children from Low- and Middle-Income Countries. Curr Trop Med Rep. 2024;11(1):28–39. doi: 10.1007/s40475-024-00314-2 38993355 PMC11238937

[13] Campbell DI, Murch SH, Elia M, Sullivan PB, Sanyang MS, Jobarteh B, et al. Chronic T cell-mediated enteropathy in rural west African children: relationship with nutritional status and small bowel function. Pediatr Res. 2003;54(3):306–11. doi: 10.1203/01.PDR.0000076666.16021.5E 12788978

[14] Kosek MN, MAL-ED Network Investigators. Causal Pathways from Enteropathogens to Environmental Enteropathy: Findings from the MAL-ED Birth Cohort Study. EBioMedicine. 2017;18:109–17. doi: 10.1016/j.ebiom.2017.02.024 28396264 PMC5405169

[15] Vielot NA, González F, Reyes Y, Zepeda O, Blette B, Paniagua M, et al. Risk Factors and Clinical Profile of Sapovirus-associated Acute Gastroenteritis in Early Childhood: A Nicaraguan Birth Cohort Study. Pediatr Infect Dis J. 2021;40(3):220–6. doi: 10.1097/INF.0000000000003015 33464013 PMC7878336

[16] Gutiérrez L, Vielot NA, Herrera R, Reyes Y, Toval-Ruíz C, Blandón P, et al. Giardia lamblia risk factors and burden in children with acute gastroenteritis in a Nicaraguan birth cohort. PLoS Negl Trop Dis. 2024;18(11):e0012230. doi: 10.1371/journal.pntd.0012230 39527625 PMC11581391

[17] Stroup SE, Roy S, Mchele J, Maro V, Ntabaguzi S, Siddique A, et al. Real-time PCR detection and speciation of Cryptosporidium infection using Scorpion probes. J Med Microbiol. 2006;55(Pt 9):1217–22. doi: 10.1099/jmm.0.46678-0 16914651

[18] Verweij JJ, Schinkel J, Laeijendecker D, van Rooyen MAA, van Lieshout L, Polderman AM. Real-time PCR for the detection of Giardia lamblia. Mol Cell Probes. 2003;17(5):223–5. doi: 10.1016/s0890-8508(03)00057-4 14580396

[19] Arndt MB, Cantera JL, Mercer LD, Kalnoky M, White HN, Bizilj G, et al. Validation of the Micronutrient and Environmental Enteric Dysfunction Assessment Tool and evaluation of biomarker risk factors for growth faltering and vaccine failure in young Malian children. PLoS Negl Trop Dis. 2020;14(9):e0008711. doi: 10.1371/journal.pntd.0008711 32997666 PMC7549819

[20] Peña R, Wall S, Persson LA. The effect of poverty, social inequity, and maternal education on infant mortality in Nicaragua, 1988-1993. Am J Public Health. 2000;90:64–9. doi: 10.2105/ajph.90.1.64 10630139 PMC1446115

[21] World Health Organization. WHO Child Growth Standards: Length/height-for-age, weight-for-age, weight-for-length, weight-forheight and body mass index-for-age: methods and development. World Heal Organ; 2006.

[22] Schober P, Boer C, Schwarte LA. Correlation Coefficients: Appropriate Use and Interpretation. Anesth Analg. 2018;126(5):1763–8. doi: 10.1213/ANE.0000000000002864 29481436

[23] Becker-Dreps S, Bucardo F, Vilchez S, Zambrana LE, Liu L, Weber DJ, et al. Etiology of childhood diarrhea after rotavirus vaccine introduction: a prospective, population-based study in Nicaragua. Pediatr Infect Dis J. 2014;33(11):1156–63. doi: 10.1097/INF.0000000000000427 24879131 PMC4216626

[24] Gutiérrez-Gutiérrez F, Palomo-Ligas L. Change in the incidence of intestinal diseases caused by parasitic protozoa in the Mexican population during the period (2015-2019) and its association with environmental and socioeconomic risk factors. Parasitol Res. 2023;122:903–14. doi: 10.1007/s00436-023-07798-3 36820929

[25] Faria CP, Zanini GM, Dias GS, da Silva S, de Freitas MB, Almendra R, et al. Geospatial distribution of intestinal parasitic infections in Rio de Janeiro (Brazil) and its association with social determinants. PLoS Negl Trop Dis. 2017;11(3):e0005445. doi: 10.1371/journal.pntd.0005445 28273080 PMC5358884

[26] Hajare ST, Betcha A, Sharma RJ, Bhosale SB, Upadhye VJ, Kuddus M, et al. Giardia lamblia infection and associated risk factors among patients attending Kochore Health Center, Ethiopia. Infect Dis Now. 2022;52(5):311–4. doi: 10.1016/j.idnow.2022.04.005 35483635

[27] Legge H, Pullan RL, Sartorius B. Improved household flooring is associated with lower odds of enteric and parasitic infections in low- and middle-income countries: A systematic review and meta-analysis. PLOS Glob Public Health. 2023;3(12):e0002631. doi: 10.1371/journal.pgph.0002631 38039279 PMC10691699

[28] Halliday KE, Kepha S, Legge H, Allen E, Dreibelbis R, Elson L, et al. Evaluating impacts of improved flooring on enteric and parasitic infections in rural households in Kenya: study protocol for a cluster-randomised controlled trial. BMJ Open. 2025;15(6):e090464. doi: 10.1136/bmjopen-2024-090464 40480665 PMC12161379

[29] Liu J, Ma’ayeh S, Peirasmaki D, Lundström-Stadelmann B, Hellman L, Svärd SG. Secreted Giardia intestinalis cysteine proteases disrupt intestinal epithelial cell junctional complexes and degrade chemokines. Virulence. 2018;9(1):879–94. doi: 10.1080/21505594.2018.1451284 29726306 PMC5955458

[30] Cascais-Figueiredo T, Austriaco-Teixeira P, Fantinatti M, Silva-Freitas ML, Santos-Oliveira JR, Coelho CH. Giardiasis Alters Intestinal Fatty Acid Binding Protein (I-FABP) and Plasma Cytokines Levels in Children in Brazil. Pathogens. 2019;9:7. doi: 10.3390/pathogens9010007 31861618 PMC7169386

[31] DeBoer MD, Scharf RJ, Leite AM, Férrer A, Havt A, Pinkerton R, et al. Systemic inflammation, growth factors, and linear growth in the setting of infection and malnutrition. Nutrition. 2017;33:248–53. doi: 10.1016/j.nut.2016.06.013 27712965 PMC5193489

[32] Goldstein S, Harp JB, Phillips LS. Nutrition and somatomedin. XXII: Molecular regulation of insulin-like growth factor-I during fasting and refeeding in rats. J Mol Endocrinol. 1991;6(1):33–43. doi: 10.1677/jme.0.0060033 2015055

[33] Trelis M, Taroncher-Ferrer S, Gozalbo M, Ortiz V, Soriano JM, Osuna A, et al. Giardia intestinalis and Fructose Malabsorption: A Frequent Association. Nutrients. 2019;11(12):2973. doi: 10.3390/nu11122973 31817420 PMC6950212

[34] Cordingley FT, Crawford GP. Giardia infection causes vitamin B12 deficiency. Aust N Z J Med. 1986;16(1):78–9. doi: 10.1111/j.1445-5994.1986.tb01127.x 3458451

[35] Bhargava A, Cotton JA, Dixon BR, Gedamu L, Yates RM, Buret AG. Giardia duodenalis Surface Cysteine Proteases Induce Cleavage of the Intestinal Epithelial Cytoskeletal Protein Villin via Myosin Light Chain Kinase. PLoS One. 2015;10(9):e0136102. doi: 10.1371/journal.pone.0136102 26334299 PMC4559405

[36] Buret A, Gall DG, Olson ME. Growth, activities of enzymes in the small intestine, and ultrastructure of microvillous border in gerbils infected with Giardia duodenalis. Parasitol Res. 1991;77(2):109–14. doi: 10.1007/BF00935423 2027878

[37] Thissen JP, Pucilowska JB, Underwood LE. Differential regulation of insulin-like growth factor I (IGF-I) and IGF binding protein-1 messenger ribonucleic acids by amino acid availability and growth hormone in rat hepatocyte primary culture. Endocrinology. 1994;134(3):1570–6. doi: 10.1210/endo.134.3.7509741 7509741

[38] Tsugawa Y, Handa H, Imai T. Arginine induces IGF-1 secretion from the endoplasmic reticulum. Biochem Biophys Res Commun. 2019;514(4):1128–32. doi: 10.1016/j.bbrc.2019.05.044 31101333

