# Supplementary material for: Natural history and impact of Giardia lamblia on child growth attainment and associated pathway-specific biomarkers in a Nicaraguan birth cohort
Source: PLoS Negl Trop Dis. 2026 May 15;20(5):e0013734. doi: 10.1371/journal.pntd.0013734 (PMC13189419; doi:10.1371/journal.pntd.0013734)
Supplement: S1 Table — (DOCX) [file pntd.0013734.s001.docx]

| **S1 Table.** Baseline epidemiological characteristics of the sub-cohort versus the remaining cohort | | | |  |  |
| --- | --- | --- | --- | --- | --- |
|  |  |  |  |  |  |
| **Characteristics** | **n (%) or median (IQR)** | | **P value*** |  | |
|  | **Children sub-cohort**  **(n=76)** | **Remaining cohort**  **(n=367)** |  |  | |
| *Birth characteristic* |  |  |  |  | |
| Sex (%Female) | 31 (40.8) | 186 (50.7) | 0.116 |  | |
| Mode of delivery (%Vaginal) | 36 (47.4) | 206 (56.1) | 0.163 |  | |
| Mother's age at birth (years) | 20 (23, 26) | 25 (22, 28) | 0.066 |  | |
| Mean birthweight (in kg) | 3.2 (3.0, 3.5) | 3.1 (2.9, 3.5) | 0.185 |  | |
| Child's HBGAs profile |  |  |  |  | |
| Lewis a-b+ | 57 (75.0) | 262 (72.0) | 0.592 |  | |
| Lewis a+b- | 3 (3.9) | 33 (9.1) | 0.170 |  | |
| Lewis a-b- | 16 (21.1) | 69 (19.0) | 0.674 |  | |
| Secretor status (%Secretor) | 71 (93.4) | 314 (86.3) | 0.125 |  | |
| *Socioeconomic and household conditions* |  |  |  |  | |
| SES (% poor or extremely poor)^α^ | 28 (37.8)^§^ | 133 (43.9)^ꓕ^ | 0.142 |  | |
| Sanitation type (%Latrine) | 21 (27.6) | 101 (27.5) | 0.984 |  | |
| Floor-type (%Earthen) | 20 (26.31) | 115 (31.3) | 0.276 |  | |
| Water resources (%Non-potable at home) | 5 (6.6) | 53 (14.4) | 0.090 |  | |
| *Nutrition* |  |  |  |  | |
| Ever Breastfeeding | 73 (96.0) | 357 (97.3) | 0.875 |  | |
| Breastfeeding durations (in months) | 17 (6, 32) | 18 (6, 32) | 0.556 |  | |
| SES: Socioeconomic status. ^α^SES was assessed using a poverty index according to Peña *et al* [20]. *Pearson's chi-square test or Fisher's exact test for cell sizes <5 for categorical variables. Mann-Whitney U test was used for numerical variables. ^§^ Two missing values. ^ꓕ^Sixty-four missing values. | | | |  |  |
|  |  |  |  |  |  |
