# Supplementary material for: Natural history and impact of Giardia lamblia on child growth attainment and associated pathway-specific biomarkers in a Nicaraguan birth cohort
Source: PLoS Negl Trop Dis. 2026 May 15;20(5):e0013734. doi: 10.1371/journal.pntd.0013734 (PMC13189419; doi:10.1371/journal.pntd.0013734)
Supplement: S2 Table — (DOCX) [file pntd.0013734.s002.docx]

| **S2 Table.** Biomarkers Associated with Growth, Systemic Inflammation, and Nutritional Status^1^ | |  |
| --- | --- | --- |
|  |  |  |
| **Indications** | **Biomarker** |  |
| Growth and Vaccine Response | Intestinal fatty acid binding protein (I-FABP) |  |
|  | Soluble CD14 (sCD14) |  |
|  | Insulin-like growth factor 1 (IGF-1) |  |
|  | Fibroblast growth factor 21 (FGF21) |  |
|  |  |  |
| Systemic Inflammation | Alpha-1-acid glycoprotein (AGP) |  |
|  | C-reactive protein (CRP) |  |
|  |  |  |
| Nutrition | Soluble transferrin receptor (sTfR) |  |
|  | Retinol binding protein 4 (RBP4) |  |

Reference: 1. Arndt MB, Cantera JL, Mercer LD, Kalnoky M, White HN, Bizilj G, et al. Validation of the Micronutrient and Environmental Enteric Dysfunction Assessment Tool and evaluation of biomarker risk factors for growth faltering and vaccine failure in young Malian children. Azman AS, editor. PLoS Negl Trop Dis. 2020;14: e0008711. doi:10.1371/journal.pntd.0008711
