# Supplementary material for: Natural history and impact of Giardia lamblia on child growth attainment and associated pathway-specific biomarkers in a Nicaraguan birth cohort
Source: PLoS Negl Trop Dis. 2026 May 15;20(5):e0013734. doi: 10.1371/journal.pntd.0013734 (PMC13189419; doi:10.1371/journal.pntd.0013734)
Supplement: S3 Table — (DOCX) [file pntd.0013734.s003.docx]

| **S3 Table.** Epidemiological characteristics of children living inside vs outside *Giardia* burden area (n=76 children). | | | | |  |  |
| --- | --- | --- | --- | --- | --- | --- |
|  |  |  |  |  |  | |
| **Characteristics** | **n (%) or median (IQR)** | | **P value*** |  | |  |
|  | **Children living inside the high burden area (n=15)** | **Children living outside high burden area (n=61)** |  |  | |  |
| *Birth characteristic* |  |  |  |  | |  |
| Sex (%Female) | 8 (53.3) | 23 (37.7) | 0.270 |  | |  |
| Mode of delivery (%Vaginal) | 7 (46.7) | 29 (47.5) | 0.952 |  | |  |
| *Socioeconomic and household conditions* |  |  |  |  | |  |
| **SES (% poor or extremely poor) *(2 missing)*α** | **9 (64.3)** | **19 (31.7)** | **0.023** |  | |  |
| **Sanitation type (%Latrine)** | **8 (53.3)** | **13 (21.3)** | **0.013** |  | |  |
| Floor-type (%Earthen) | 5 (33.3) | 15 (24.6) | 0.491 |  | |  |
| Water resources (%Non-potable at home) | 0 (0.0) | 5 (8.2) | 0.251 |  | |  |
| AGE: acute gastroenteritis. SES: Socioeconomic status. αSES was assessed using a poverty index according to Peña *et al* [20]. *Pearson's chi-square test or Fisher's exact test for cell sizes <5 for categorical variables. Mann-Whitney U test was used for numerical variables | | | | |  | |
|  |  |  |  |  |  | |
