# Supplementary material for: Natural history and impact of Giardia lamblia on child growth attainment and associated pathway-specific biomarkers in a Nicaraguan birth cohort
Source: PLoS Negl Trop Dis. 2026 May 15;20(5):e0013734. doi: 10.1371/journal.pntd.0013734 (PMC13189419; doi:10.1371/journal.pntd.0013734)
Supplement: S4 Table — (DOCX) [file pntd.0013734.s004.docx]

| **S4 Table**. β-estimated coefficient of linear regression using GEE on child anthropometric indicators in children infected with *Giardia* (n=44). | | | | | |  |
| --- | --- | --- | --- | --- | --- | --- |
|  |  |  |  |  |  |  |
| Category | | β | IC95% | | P value |  |
|  |  |  | Higher | Lower |  |  |
| LAZ | **Any *Giardia* (n=44)** | **-0.16** | **-0.01** | **-0.32** | **0.042** |  |
|  | Any Persistent *Giardia* (n=18) | -0.11 | 0.03 | -0.26 | 0.134 |  |
|  | **Any Recurrent *Giardia* (n=20)** | **-0.37** | **-0.19** | **-0.56** | **0.000** |  |
|  | **Any Persistent/Recurrent (n=28)** | **-0.26** | **-0.11** | **-0.41** | **0.001** |  |
|  | Less than 2 infections (n=23) | NA | NA | NA | NA |  |
|  | **More than 2 infections (n=21)** | **-0.30** | **-0.14** | **-0.45** | **0.000** |  |
|  | **1^st^ infection before 18 months (n=12)** | **-0.24** | **-0.02** | **-0.44** | **0.028** |  |
|  | 1^st^ infection after 18 months (n=32) | -0.08 | 0.14 | -0.31 | 0.465 |  |
|  | **Children in High-*Giardia* area (n=10)** | **-0.44** | **-0.18** | **-0.70** | **0.001** |  |
|  | **Children in not High-*Giardia* area (n=34)** | **-0.19** | **-0.03** | **-0.35** | **0.022** |  |
|  |  |  |  |  |  |  |
| WAZ | Any *Giardia* (n=44) | -0.01 | 0.09 | -0.12 | 0.787 |  |
|  | Any Persistent *Giardia* (n=18) | 0.03 | 0.23 | -0.17 | 0.753 |  |
|  | Any Recurrent *Giardia* (n=20) | 0.02 | 0.14 | -0.11 | 0.801 |  |
|  | Any Persistent/Recurrent (n=28) | 0.04 | 0.17 | -0.10 | 0.588 |  |
|  | Less than 2 infections (n=23) | NA | NA | NA | NA |  |
|  | More than 2 infections (n=21) | -0.02 | 0.16 | -0.21 | 0.796 |  |
|  | 1^st^ infection before 18 months (n=12) | 0.02 | 0.16 | -0.12 | 0.786 |  |
|  | 1^st^ infection after 18 months (n=32) | -0.07 | 0.07 | -0.22 | 0.306 |  |
|  | Children in High-*Giardia* area (n=10) | -0.17 | 0.08 | -0.41 | 0.175 |  |
|  | Children in not High-*Giardia* area (n=34) | 0.02 | 0.16 | -0.13 | 0.841 |  |
|  |  |  |  |  |  |  |
| WFL/H | Any *Giardia* (n=44) | 0.11 | 0.29 | -0.07 | 0.218 |  |
|  | Any Persistent *Giardia* (n=18) | 0.09 | 0.42 | -0.24 | 0.587 |  |
|  | Any Recurrent *Giardia* (n=20) | 0.24 | 0.51 | -0.02 | 0.072 |  |
|  | **Any Persistent/Recurrent (n=28)** | **0.24** | **0.44** | **0.04** | **0.019** |  |
|  | Less than 2 infections (n=23) | NA | NA | NA | NA |  |
|  | **More than 2 infections (n=21)** | **0.24** | **0.43** | **0.05** | **0.015** |  |
|  | 1^st^ infection before 18 months (n=12) | 0.22 | 0.48 | -0.04 | 0.091 |  |
|  | 1^st^ infection after 18 months (n=32) | -0.02 | 0.20 | -0.26 | 0.815 |  |
|  | Children in High-*Giardia* area (n=10) | 0.05 | 0.50 | -0.41 | 0.840 |  |
|  | Children in not High-*Giardia* area (n=34) | 0.15 | 0.39 | -0.10 | 0.251 |  |
| The β-estimated coefficient was calculated for length-for-age (LAZ), weight-for-age (WAZ), and weight-for-length/height (WFL/H) Z-scores using generalized estimating equations (GEE), and the value one month prior to the *Giardia* infection was used as the baseline. Each model was adjusted for age, mode of delivery, sex, socioeconomic status, breastfeeding, and episodes of diarrhea. Any recurrent infection was defined if a child had more than one *Giardia*-positive qPCR result in non-consecutive stool samples. Any persistent *Giardia* infections were defined if two or more consecutive routine stool samples that tested positive for *Giardia*. Children living within or outside high-*Giardia* burden areas based on the Kernel density distribution of *Giardia* infections. | | | | | |  |
|  |  |  |  |  |  |  |
|  |  |  |  |  |  |  |
|  |  |  |  |  |  |  |
|  |  |  |  |  |  |  |
|  |  |  |  |  |  |  |
|  |  |  |  |  |  |  |
