# Supplementary material for: Natural history and impact of Giardia lamblia on child growth attainment and associated pathway-specific biomarkers in a Nicaraguan birth cohort
Source: PLoS Negl Trop Dis. 2026 May 15;20(5):e0013734. doi: 10.1371/journal.pntd.0013734 (PMC13189419; doi:10.1371/journal.pntd.0013734)
Supplement: S5 Table — (DOCX) [file pntd.0013734.s005.docx]

| **S5 Table.** Fecal biomarkers measured at 24 and 36 months of age in children infected at least once with *Giardia* infections and children not infected. | | | | |  |
| --- | --- | --- | --- | --- | --- |
|  |  |  |  |  |  |
|  | **Biomarkers** | ***Giardia**** | **No *Giardia**** | **P value^ꓕ^** |  |
|  |  |  |  |  |  |
| at 24M | MPO | 1.46 (0.98-1.92) | 1.49 (0.31-2.21) | 0.947 |  |
|  | NEO | 255.61 (127.95-634.92) | 250.67 (166.67-457.71) | 0.978 |  |
|  | REG-1β | 92.57 (29.85-301.53) | 139.67 (91.84-367.15) | 0.183 |  |
|  |  |  |  |  |  |
| at 36M | MPO | 0.93 (0.39-2.55) | 0.45 (0.19-1.53) | 0.074 |  |
|  | **NEO** | **209.1 (127.15-290.25)** | **355.4 (184.75-1050.05)** | **0.023** |  |
|  | REG-1β | 136.2 (35.01-191.3) | 219.46 (27.91-354.6) | 0.233 |  |
| Fecal biomarkers measured at 24 (24M) and 36 months of age (36M) in children infected at least once with *Giardia* infections at 24M (*Giardia*, n=24) and 36M (n=31), and children not infected at 24M (No *Giardia*, n=34) and 36M (n=27) for: Myeloperoxidase (MPO), Neopterin (NEO), and Regenerating family member 1β (Reg-1β). *Median (IQR). ^ꓕ^Mann-Whitney U test was used for numerical variables | | | | |  |
|  |  |  |  |  |  |
|  |  |  |  |  |  |
|  |  |  |  |  |  |
