# Supplementary material for: Natural history and impact of Giardia lamblia on child growth attainment and associated pathway-specific biomarkers in a Nicaraguan birth cohort
Source: PLoS Negl Trop Dis. 2026 May 15;20(5):e0013734. doi: 10.1371/journal.pntd.0013734 (PMC13189419; doi:10.1371/journal.pntd.0013734)
Supplement: S6 Table — (DOCX) [file pntd.0013734.s006.docx]

| **S6 Table.** Systemic biomarkers measured at 24 months of age in children infected at least once with *Giardia* infections (*Giardia*, n=29) and children not infected (No *Giardia*, n=20). | | | |  |
| --- | --- | --- | --- | --- |
|  |  |  |  |  |
| **Biomarkers** | **Children with *Giardia**** | **Children without *Giardia**** | **P value^ꓕ^** |  |
|  |  |  |  |  |
| **I-FABP (pg/ml)** | **7074.8 (4271.4-11085.8)** | **4573.0 (1807.4-5823.7)** | **0.002** |  |
| **IGF-1 (ng/ml)** | **0.1 (0.1-31.3)** | **203.3 (0.1-433.6)** | **0.005** |  |
| **Anti-FliC IgA (ng/L)** | **9.8 (7.9-11.2)** | **8.2 (6.9-9.1)** | **0.033** |  |
| AGP (g/L) | 10.5 (7.8-14.9) | 9.9 (6.3-13.2) | 0.186 |  |
| RBP4 (umol/L) | 16.8 (14.9-19.6) | 19.1 (14.9-21.6) | 0.231 |  |
| sCD14 (ng/ml) | 19204.3 (14659.9-23929.8) | 18110.2 (9184.3-21173.3) | 0.271 |  |
| FGF21 (pg/ml) | 1372.2 (720.8-2254.6) | 1001.8 (443.7-2035.4) | 0.402 |  |
| CRP (mg/L) | 4.8 (1.6-15.5) | 2.3 (0.7-30.5) | 0.469 |  |
| sTfR (mg/L) | 1081.8 (630.7-1283.4) | 1174.6 (640.3-1532.3) | 0.512 |  |
| Anti-flagellin C (Anti-FliC) -IgA.Insulin-like growth factor-1 (IGF-1). Intestinal fatty acid binding protein (I-FABP). Fibroblast growth factor 21 (FGF21). Soluble transferrin receptor (sTfR). C-reactive protein (CRP). Retinol binding protein 4 (RBP4). α-1-acid glycoprotein (AGP). Soluble cluster of differentiation 14 (sCD14). *Median (IQR). ^ꓕ^Mann-Whitney U test was used for numerical variables | | | |  |
|  |  |  |  |  |
|  |  |  |  |  |
|  |  |  |  |  |
