# Supplementary material for: Natural history and impact of Giardia lamblia on child growth attainment and associated pathway-specific biomarkers in a Nicaraguan birth cohort
Source: PLoS Negl Trop Dis. 2026 May 15;20(5):e0013734. doi: 10.1371/journal.pntd.0013734 (PMC13189419; doi:10.1371/journal.pntd.0013734)
Supplement: S7 Table — (DOCX) [file pntd.0013734.s007.docx]

| **S7 Table.** β-estimates from linear regression models of log2-transformed fecal and systemic biomarker concentrations on child anthropometric indicators at 24 months of age. | | | | | | |  |
| --- | --- | --- | --- | --- | --- | --- | --- |
|  |  |  |  |  |  |  |  |
|  |  |  |  |  |  |  |  |
| Indicator | Biomarkers | Type | β-estimateꓕ | IC95% | | P value |  |
|  |  |  |  | Lower | Higher |  |  |
| LAZ | **AGP** | **Systemic** | **-0.694** | **-1.250** | **-0.134** | **0.016** |  |
|  | Anti FliC-IgA | Systemic | 0.428 | -0.622 | 1.480 | 0.413 |  |
|  | CRP | Systemic | -0.081 | -0.188 | 0.026 | 0.133 |  |
|  | FGF-21 | Systemic | -0.088 | -0.200 | 0.023 | 0.117 |  |
|  | **I-FABP** | **Systemic** | **-0.273** | **-0.538** | **-0.008** | **0.044** |  |
|  | **IGF-1** | **Systemic** | **0.070** | **0.005** | **0.136** | **0.036** |  |
|  | MPO | Fecal | 0.079 | -0.046 | 0.205 | 0.209 |  |
|  | NEO | Fecal | 0.017 | -0.213 | 0.247 | 0.884 |  |
|  | RBP4 | Systemic | -0.171 | -0.980 | 0.639 | 0.672 |  |
|  | REG-1β | Fecal | 0.042 | -0.096 | 0.181 | 0.544 |  |
|  | sCD14 | Systemic | -0.090 | -0.269 | 0.088 | 0.311 |  |
|  | sTfR | Systemic | 0.094 | -0.492 | 0.680 | 0.746 |  |
|  |  |  |  |  |  |  |  |
| WAZ | AGP | Systemic | -0.448 | -0.903 | 0.008 | 0.054 |  |
|  | Anti FliC-IgA | Systemic | -0.143 | -0.932 | 0.645 | 0.713 |  |
|  | CRP | Systemic | -0.053 | -0.138 | 0.033 | 0.218 |  |
|  | FGF-21 | Systemic | -0.028 | -0.119 | 0.062 | 0.525 |  |
|  | I-FABP | Systemic | -0.135 | -0.353 | 0.083 | 0.219 |  |
|  | IGF-1 | Systemic | 0.049 | -0.003 | 0.102 | 0.064 |  |
|  | MPO | Fecal | 0.059 | -0.033 | 0.150 | 0.204 |  |
|  | **NEO** | **Fecal** | **0.168** | **0.007** | **0.329** | **0.041** |  |
|  | RBP4 | Systemic | 0.170 | -0.469 | 0.810 | 0.592 |  |
|  | REG-1β | Fecal | -0.053 | -0.154 | 0.047 | 0.289 |  |
|  | sCD14 | Systemic | -0.054 | -0.196 | 0.088 | 0.445 |  |
|  | sTfR | Systemic | 0.143 | -0.319 | 0.604 | 0.535 |  |
|  |  |  |  |  |  |  |  |
| WFL | AGP | Systemic | -0.106 | -0.636 | 0.423 | 0.686 |  |
|  | Anti FliC-IgA | Systemic | -0.529 | -1.340 | 0.283 | 0.194 |  |
|  | CRP | Systemic | -0.013 | -0.110 | 0.083 | 0.780 |  |
|  | FGF-21 | Systemic | 0.025 | -0.076 | 0.126 | 0.617 |  |
|  | I-FABP | Systemic | 0.022 | -0.227 | 0.271 | 0.859 |  |
|  | IGF-1 | Systemic | 0.016 | -0.045 | 0.077 | 0.595 |  |
|  | MPO | Fecal | 0.020 | -0.084 | 0.124 | 0.698 |  |
|  | **NEO** | **Fecal** | **0.225** | **0.049** | **0.402** | **0.013** |  |
|  | RBP4 | Systemic | 0.363 | -0.337 | 1.060 | 0.300 |  |
|  | REG-1β | Fecal | -0.105 | -0.214 | 0.005 | 0.061 |  |
|  | sCD14 | Systemic | -0.007 | -0.166 | 0.151 | 0.928 |  |
|  | sTfR | Systemic | 0.118 | -0.394 | 0.630 | 0.643 |  |
| ꓕβ-estimates from multiple linear regression models of fecal and systemic biomarkers on length-for-age (LAZ), weight-for-age (WAZ), and weight-for-length (WFL) Z-scores at 24 months of age, adjusted for mode of delivery, sex, socioeconomic status, breastfeeding, and episodes of diarrhea. A total of 36 separate models were run (one model per biomarker for each anthropometric indicator. Fecal (n=56) and systemic biomarker (n=49). Anti-flagellin C (Anti-FliC) -IgA. Insulin-like growth factor-1 (IGF-1). Intestinal fatty acid binding protein (I-FABP). Fibroblast growth factor 21 (FGF21). Soluble transferrin receptor (sTfR). C-reactive protein (CRP). Retinol binding protein 4 (RBP4). α-1-acid glycoprotein (AGP). Soluble cluster of differentiation 14 (sCD14), Myeloperoxidase (MPO), Neopterin (NEO), Regenerating family member 1β (Reg-1β). | | | | | | |  |
|  |  |  |  |  |  |  |  |
|  |  |  |  |  |  |  |  |
|  |  |  |  |  |  |  |  |
|  |  |  |  |  |  |  |  |
|  |  |  |  |  |  |  |  |
|  |  |  |  |  |  |  |  |
